# Strong Teams, Strong Systems: Rethinking Aid for Global Surgery

**DOI:** 10.1002/wjs.70382

**Published:** 2026-04-25

**Authors:** Sugy Choi, Jayoung Park, Woong‐Han Kim, Andualem Beyene, Jerel Ezell

**Affiliations:** ^1^ Department of Population Health New York University Grossman School of Medicine New York New York USA; ^2^ Program in Global Surgery and Implementation Science JW LEE Center for Global Medicine Seoul National University College of Medicine Kidney Research Institute Seoul National University Medical Research Center Seoul South Korea; ^3^ Department of Human Systems Medicine Seoul National University College of Medicine Seoul South Korea; ^4^ Department of Thoracic and Cardiovascular Surgery Seoul National University Children's Hospital, Seoul National University College of Medicine Seoul South Korea; ^5^ Department of Surgery School of Medicine College of Health Sciences Addis Ababa University Addis Ababa Ethiopia; ^6^ Community Health Science School of Public Health University of California Berkeley Berkeley California USA; ^7^ Berkeley Center for Cultural Humility University of California Berkeley Berkeley California USA

Global surgery has become a priority in international health agendas, with donor countries and aid agencies investing in infrastructure, equipment, and short‐term missions [1]. These efforts have saved lives, but they have also exposed a persistent need: Strong surgical teams do not emerge from hardware alone. They are built through relationships, trust, and shared learning. If aid is to deliver sustainable impact, it must pivot from episodic interventions toward team building grounded in cultural humility. Cultural humility, defined as a lifelong commitment to self‐evaluation and critique, redressing power imbalances, and fostering affirming partnerships, offers a lens to understand and address the complexities of building surgical teams and health systems strengthening in low‐ and middle‐income countries’ (LMICs’) settings [2].

Surgery is a team endeavor. Surgeons, anesthesiologists, nurses, perfusionists, and rehabilitation specialists must work in sync. In resource‐constrained settings, where staffing and infrastructure are thinly stretched, the margin for error narrows. Yet, donor strategies often prioritize equipment and visiting surgeons over the ecosystem of collaboration that makes care safe. In this commentary, we seek to share some of the lessons learned through past decades of collaborations, through bilateral aid, grant funding, and international partnerships aimed at expanding surgical care in LMICs.

Short‐term missions save lives but rarely build systems, and episodic interventions often bypass local governance and leave gaps in continuity. Although surgical missions can address urgent needs, they risk reinforcing dependency unless paired with long‐term capacity‐building strategies. Unfortunately, training individuals is not enough. Fellowship programs for surgeons and anesthesiologists are vital, but without team‐based routines and leadership development, skills remain siloed and fragile. Individuals continue to face barriers that are deeply embedded in systemic issues, barriers that are nearly impossible to solve alone. These include fragmented referral pathways, provider incentives, and out‐of‐pocket costs.

## 1 A Need for Culturally Humble Approaches to Global Surgery

Congenital Heart Disease (CHD) is a pressing yet under‐addressed public health issue in LMICs. Although interventions such as neonatal and pediatric open‐heart surgery offer lifesaving potential, they remain largely inaccessible due to structural barriers: inadequate infrastructure, shortages of skilled providers, and high out‐of‐pocket costs [3]. However, these barriers are not only logistical, they are also deeply social and relational, shaping how families perceive, seek, and receive care. Caregivers, often mothers, face immense financial and logistical burdens alongside emotional distress, uncertainty, and culturally specific and frequently gendered expectations around grieving, caregiving, and medical authority [4]. These realities demand a culturally humble stance—one that problematizes and resists the imposition of Global North–centric norms and instead centers the lived experiences and agencies of caregivers navigating opaque and fragmented systems.

This lens is equally critical within the pediatric cardiac surgery ecosystem itself, where complex, team‐based care models are essential. Applying cultural humility within surgical teams, through mutual respect, shared learning, and attention to local knowledge, roles, and hierarchies, can strengthen collaboration across lines of formal education and training, discipline, and culture, ultimately improving outcomes for children with CHD. Cultural humility, in this context, involves more than empathy. It requires interrogating how health systems may (inadvertently) produce or deny eligibility for care. Families' decisions and behaviors are shaped not by “compliance” or “literacy” alone, but by precarity, mistrust, prior exclusions, and deep‐rooted understandings of illness and healing.

## 2 Surgical Teams as Systems: Embedding Cultural Humility in Team‐Based Care

High‐quality pediatric cardiac care depends on well‐coordinated, multidisciplinary teams—including pediatric cardiologists, cardiothoracic surgeons, anesthesiologists, intensivists, nurses, perfusionists, and rehabilitation specialists [5]. In resource‐constrained settings, where infrastructure and staffing may be thinly stretched, the importance of cohesive team–based care becomes even more pronounced.

Cultural humility within these teams is critical. It calls for mutual respect, shared learning, shared decision‐making and attention to local knowledge and hierarchies [6]. When surgical teams operate with cultural humility—across lines of training, nationality, and culture—they can foster more sustainable collaboration, strengthen local capacity, and improve outcomes for children with CHD [7]. Furthermore, cultural humility supports the decolonization of global surgery by empowering local ownership, valuing cultural practices, and avoiding the replacement or marginalization of partners in LMICs [8].

Cultural humility is not an optional soft skill: it is a structural principle. Defined as a lifelong commitment to self‐reflection, power sharing, and affirmational partnerships, cultural humility reframes global surgery from a technical intervention to a relational enterprise. Applied to surgical teams, cultural humility means elevating local leadership in governance and decision‐making, recognizing that expertise flows both ways, and designing routines that reflect local norms and constraints. Practical steps include preoperative huddles and postoperative debriefs that integrate interpreters and personal and professional expectations, task‐sharing frameworks that elevate nursing and anesthesia roles, and career ladders for allied health professionals to strengthen retention. These practices are not cosmetic; they are structural. They determine whether a team can function under pressure, whether communication failures lead to harm, and whether local professionals feel respected and empowered.

## 3 From Missions to Sustainable Teams

Future aid must fund team ecosystems, not just missions. This requires supporting multidisciplinary training programs and leadership development for nurses, anesthetists, and allied health professionals, not only surgeons. It also means investing in maintenance and biomedical engineering capacity to keep systems functional beyond the life of a grant, requiring authorship equity and compensating local leaders for governance and research roles, and embedding transition plans to prevent dependency on external teams. Donor metrics should evolve as well. Instead of counting operations performed during a mission, success should be measured by whether 5 years later, a local team can deliver safe, timely, and affordable surgery without external dependence.

## Author Contributions

**Sugy Choi:** conceptualization, writing – original draft. **Jayoung Park:** writing – review and editing, writing – original draft. **Woong‐Han Kim:** writing – review and editing. **Andualem Beyene:** writing – review and editing. **Jerel Ezell:** conceptualization, writing – review and editing.

## Conflicts of Interest

The authors declare no conflicts of interest.

## Data Availability

Data sharing not applicable to this article as no datasets were generated or analyzed during the current study.
